# Stature and sex estimation auricular morphometry and morphology

**DOI:** 10.6026/973206300221270

**Published:** 2026-02-28

**Authors:** Pankaj Mahawar, Ameet Julka, Arpan Dubey, Shruti Tomar

**Affiliations:** 1 Department of Anatomy, Dr. Laxminarayan Pandey Government Medical college, Ratlam, Madhya Pradesh, India; 2 Department of Anatomy, M.G.M. Medical College, Indore, Madhya Pradesh, India

**Keywords:** External ear morphometry, sexual dimorphism

## Abstract

External ear morphometry is increasingly explored for forensic identification because ear dimensions show population variation and sexual dimorphism, requiring region-specific reference data. A cross-sectional study was conducted on 200 Central Indian adults (18-30 years) to assess external ear morphometry, morphology and sexual dimorphism and to explore relevance for identification purposes. Bilateral ear and lobular measurements were taken using a digital Vernier caliper and stature/weight was recorded using standard instruments. Males had significantly higher mean stature and weight than females. Ear length, ear width and lobule width were significantly greater in males on both sides, while ear index, lobule height and lobule index did not differ significantly by sex; oval ear shape predominated and Darwin's tubercle showed a significant sex difference only on the right side.

## Background:

External ear morphology and morphometry are stable anthropometric characteristics increasingly investigated for person identification in clinical and criminal settings [1]. As the auricle varies significantly across sex, age and ethnicity, population-specific databases are required for accurate interpretation and application in forensic contexts [2]. The external ear's high visibility and unique features, such as shape and lobule attachment, provide reliable secondary evidence for identification when primary physical markers are compromised. Studies highlight that ear morphology is varied enough to justify discriminatory assessment when used alongside metric parameters [3]. Sexual dimorphism is a key aspect of biological profiling, with males generally possessing larger ear dimensions than females [4]. In North Indian samples, physiognomic ear length, breadth and tragal distances have been shown to differ significantly between sexes, aiding in sex determination [5]. Research into ear height and width further documents these sex-specific variations, providing strong support for the utility of external ear anthropometry [6]. However, evidence suggests that ear size depends heavily on demographic determinants; meaning data cannot be transposed across different populations without rigorous validation. Stature estimation remains a fundamental component of the biological profile in forensic anthropology and disaster victim identification [7]. In instances where standard skeletal remains are missing, researchers utilize the positive correlation between ear dimensions and height to estimate stature through regression equations [1, 8]. Therefore, it is of interest to investigate external ear morphometry and morphology in the target population to establish sex-specific markers and validate ear measurements as a reliable, non-invasive supplement for stature estimation using population-specific analysis tools.

## Materials and Methods:

## Study design and setting:

The study is a cross-sectional one, carried out in the Department of Anatomy, Mahatma Gandhi Memorial Medical College and Maharaj Yeshwantrao Hospital, Indore, Madhya Pradesh, during a one-year study period after receiving the consent of the Institutional Scientific and Ethics Committee. Informed consent was taken through the written consent of all the participants before enrolment.

## Population and sampling of study:

The sample consisted of 200 adult students (male and female) between the ages of 18 and 30 years and studying or in employment at the institution and were individuals living in Central India. When it came to anthropometric analysis, the participants were recruited in both academic and workplace to provide a heterogenous sample, albeit age-limited, to conduct the study.

## Eligibility criteria:

The inclusion criteria were males and females between 18-30 years, living in Central India and willing to participate and sign written informed consent. The inclusion criteria were craniofacial trauma history, ear disease, ear deformities or other anomalies, ear surgery history and non-consenting participants.

## Study instruments:

Linear measurements (mm) of the ears and lobes were done using a digital Vernier caliper. Stature (cm) was measured by the use of aaudiometer/height scale. Body weight (kg) was measured using a digital weighing machine.

## Data collection procedure:

The participants were observed in a bright room in a seated position after explaining the objectives and procedures. The head was placed in Frankfurt plane to normalize the orientation when conducting measurements on the ears. Digital Vernier caliper was used to measure linear values bilaterally and all the values were captured instantly in the participant proforma.

## Ear morphometry parameters:

External ear measures were done on both sides. The length of the ear (EL) was the vertical distance between the helix highest and lobule lowest point. The ear width (EW) was the greatest distance between the root of the ear and the most protruded point of the helix. The ear index (EI) was determined by dividing ear width by ear length multiplied by 100. The height of the lobules (LH) was measured as the distance between the lower point of attachment of ear to the head and caudal endpoint of the free lobular margin. The width of ear lobules (LW) was taken as the limit of the widest transverse length of the ear lobule between its connections with the most peripheral margin. Lobule index (LI) was determined as lobule width/lobule height/ 100.

## Ear morphology assessment:

Morphological characteristics were evaluated by eye and enlisted as nominal variables. The presence or absence of the tubercle of the ear weighed on each side was reported in Darwin (auricular). Shape of ear was listed as round, oval, rectangle or triangular. Shape of ear lobule was classified as arched, triangular, tongue or square. The lobule attachment was divided into attached, partly attached and free.

## Anthropometry:

Stature was determined and the participant was in an erect posture and audiometer was used with the patient standing barefoot and with his back to an audiometer. Mass was measured in a digital weighing machine with the minimum of clothing and no footwear.

## Results and Discussion:

The study included 200 participants, comprising 106 (53.0%) males and 94 (47.0%) females (Table 1). Anthropometric analysis revealed expected sexual dimorphism in body size, with males exhibiting significantly higher mean stature (height: 174.56±6.96 cm vs 160.78±5.40 cm; weight: 62.35±11.46 kg vs 53.42±9.64 kg) compared to females (Table 2). This significant difference in overall body size is relevant as it may influence auricular dimensions. Analysis of ear morphometry revealed clear sexual dimorphism. Males demonstrated significantly greater ear length and ear width than females on both the right (p = 0.0001) and left sides (p = 0.0001) (Table 3 & Table 4). This aligns with findings by Rani *et al.* [4] in a North Indian population, supporting the consensus that absolute ear dimensions are reliable sex-discriminating variables. Interestingly, while linear dimensions differed, the Ear Index (EI) did not show significant differences between sexes on either side (p > 0.05). This indicates that while male ears are larger, the proportion of width to length remains similar across sexes, a finding consistent with Japatti *et al.* [3], who noted that indices often show less discriminatory power than raw linear measures. Regarding lobular parameters, results were mixed. Lobule width was significantly greater in males on both the right (p = 0.0001) and left sides ($p = 0.047$). However, lobule height and lobule index showed no statistically significant differences. This pattern supports observations by Acharya *et al.* [2], suggesting that specific parameters like ear length, width and lobule width are more effective for predictive modeling than lobule height. Morphological assessment showed that an oval ear shape was the predominant phenotype in both sexes and on both sides, with no statistically significant difference in distribution (Table 5). This suggests that ear shape is less useful for sex determination compared to metric parameters. However, the presence of Darwin's tubercle showed significant asymmetry and sexual dimorphism; it was significantly more common in females (37.2%) than males (20.8%) on the right side (p = 0.010), while the left side showed no significant difference (Table 6). This right-sided female predominance highlights the importance of considering asymmetry in forensic anthropometry, though it should be interpreted with caution as a population-specific trait. The strong correlation between sex and ear dimensions in this Central Indian population validates the use of ear morphometry in forensic biological profiling. Since stature estimation is a primary goal of such anthropometric data, the significant linear relationships found here-particularly in ear length and width-suggest these parameters are suitable for developing regression models for stature estimation, as proposed in similar algorithmic approaches by Acharya *et al.* [2] and Dinakaran *et al.* [8]. Future analysis of this dataset will focus on generating regression equations using the most discriminatory variables identified here (ear length, ear width and lobule width).

## Conclusion:

Central Indian adults exhibit significant sexual dimorphism in ear size, with males possessing larger dimensions, though proportional indices remain similar. While ear morphology is largely comparable between sexes, the findings suggest that metric dimensions are primary for sex determination, while morphological traits like Darwin's tubercle serve as valuable supportive evidence.

## Figures and Tables

**Table 1:** Participant distribution by sex (N = 200)

**Sex**	**N**	**%**
Male	106	53
Female	94	47
Total	200	100

**Table 2:** Stature and weight by sex

**Variable**	**Male (Mean±SD)**	**Female (Mean±SD)**
Height (cm)	174.56±6.96	160.78±5.40
Weight (kg)	62.35±11.46	53.42±9.64

**Table 3:** Lexternal ear morphometry by sex (right ear)

**Parameter**	**Male (Mean±SD)**	**Female (Mean±SD)**	**p-value**
Right ear length (mm)	63.58±3.96	59.59±3.45	0.0001
Right ear width (mm)	35.00±2.19	32.48±2.31	0.0001
Right ear index	55.18±3.9	54.02±6.9	0.271
Right lobule height (mm)	18.60±2.46	18.12±2.12	0.146
Right lobule width (mm)	21.00±2.76	19.89±2.73	0.0001
Right lobule index	114±18.05	110.76±16.16	0.154

**Table 4:** External ear morphometry by sex (Left ear)

**Parameter**	**Male (Mean±SD)**	**Female (Mean±SD)**	**p-value**
Left ear length (mm)	63.26±3.66	59.40±3.32	0.0001
Left ear width (mm)	34.30±4.25	31.98±3.85	0.0001
Left ear index	54.32±6.7	53.88±6.7	0.638
Left lobule height (mm)	18.56±2.66	18.25±2.09	0.334
Left lobule width (mm)	20.91±2.56	20.19±2.47	0.047
Left lobule index	114.9±18.6	111.71±15.69	0.278

**Table 5:** Ear morphology (shape) by sex

**Side**	**Shape**	**Male n (%)**	**Female n (%)**	**Total n (%)**	**p-value**
Right ear	Oval	72 (67.9)	55 (58.5)	127 (63.5)	0.244
Right ear	Rectangular	29 (27.4)	36 (38.3)	65 (32.5)	
Right ear	Triangular	5 (4.7)	3 (3.2)	8 (4.0)	
Right ear	Round	0 (0.0)	0 (0.0)	0 (0.0)	
Left ear	Oval	71 (67.0)	68 (72.3)	139 (69.5)	0.141
Left ear	Rectangular	26 (24.5)	23 (24.5)	49 (24.5)	
Left ear	Triangular	9 (8.5)	2 (2.1)	11 (5.5)	
Left ear	Round	0 (0.0)	1 (1.1)	1 (0.5)	

**Table 6:** Darwin's tubercle (presence) by sex

**Side**	**Darwin's tubercle**	**Male n (%)**	**Female n (%)**	**Total n (%)**	**p-value**
Right	Present	22 (20.8)	35 (37.2)	57 (28.5)	0.01
Right	Absent	84 (79.2)	59 (62.8)	143 (71.5)	
Left	Present	25 (23.6)	21 (22.3)	46 (23.0)	0.835
Left	Absent	81 (76.4)	73 (77.7)	154 (77.0)	
